# Metagenome-Assembled Genome of USCα AHI, a Potential High-Affinity Methanotroph from Axel Heiberg Island, Canadian High Arctic

**DOI:** 10.1128/MRA.01178-19

**Published:** 2019-11-14

**Authors:** Calvin Rusley, Tullis C. Onstott, Tatiana A. Vishnivetskaya, Alice Layton, Archana Chauhan, Susan M. Pfiffner, Lyle G. Whyte, Maggie C. Y. Lau

**Affiliations:** a Department of Geosciences, Princeton University, Princeton, New Jersey, USA; b Center for Environmental Biotechnology, University of Tennessee, Knoxville, Tennessee, USA; c Department of Natural Resource Sciences, McGill University, Montreal, Québec, Canada; University of Southern California

## Abstract

Metagenomic sequencing of active-layer cryosols from the Canadian High Arctic has yielded a nearly complete genome for an atmospheric CH_4_-oxidizing bacterium belonging to upland soil cluster α (USCα). This genome contains genes involved in CH_4_ metabolism, H_2_ metabolism, and multiple carbon assimilation pathways.

## ANNOUNCEMENT

Recent studies have shown that mineral cryosols from the Canadian High Arctic Axel Heiberg Island (AHI) act as CH_4_ sinks during the summer (1), drawing CH_4_ from both the atmosphere and underlying hypoxic cryosols (2, 3), and harbor metabolically active upland soil cluster α (USCα) proteobacteria (1). Twenty-one metagenomic data sets of active-layer cryosols (4) from long-term core incubation experiments were used to construct the draft genome of this USCα. Sequencing and sample collection methods were published by Chauhan et al. (4).

Raw reads were filtered using the Princeton University Galaxy server using “filter by quality” to keep reads having 90% of the bases with a Phred score of >30. Nextera transposase adaptor sequences and the last five bases at the 3′ end were removed using Trim Galore. IDBA-UD v1.1.1 (with the settings mink = 20, maxk = 100, and step = 20) was used to create 21 individual assemblies and 1 coassembly from reads longer than 50 nucleotides (nt) (5). Bins were created using MetaBAT v0.32.4 (6) (–very sensitive option), evaluated using CheckM v1.0.6 (7), and annotated using PROKKA v1.12-beta (8) and BLAST v2.2.29+ (9). Default parameters were used for all software unless otherwise specified. The coassembly yielded a 90.56% complete genome with 0.31% contamination, containing a USCα-like particulate methane monooxygenase β-subunit (*pmoA*) gene. CheckM assigned this genome as an unknown species within the *Beijerinckiaceae*.

As CheckM analysis indicated that 4 of the 21 individual assemblies had unknown *Beijerinckiaceae* bins (6.43 to 36.49% complete), we extracted *Beijerinckiaceae* reads from these 4 metagenomes (SRA accession numbers SRR1586250, SRR1586265, SRR1586287, and SRR1586310). We then mapped the quality-filtered reads onto the USCα bin and four *Beijerinckiaceae* genomes having different phylogenetic distances from USCα (10), namely, Methylocapsa acidiphila B2 (NZ_ATYA01000001), Methylocella silvestris BL2 (NC_011666), *Methylocystis* sp. strain SC2 (NC_018485), and Methylosinus trichosporium OB3b (NZ_ADVE02000003), using Bowtie2 v2.3.2 (11). All mapped reads were pooled and reassembled using SPAdes v3.10.1 (12). Binning using MetaBAT v0.32.4 (–very sensitive option) yielded a single bin. Evaluated by CheckM v1.0.6, this final genome had slightly improved completeness and less contamination (Table 1). This genome was annotated using PROKKA v1.12-beta (8), BLAST v2.2.29+ (9) against the SILVA SSU v128 and NCBI databases, and the Kyoto Encyclopedia of Genes and Genomes (KEGG) automatic annotation server v2.1 (13). A phylogenetic tree using single-copy genes (14) was created using Anvi’o v5.2 (15) phylogenomic analysis for *Beijerinckiaceae* genomes selected by referencing Tveit et al. (10). Average nucleotide identity (ANI) and average amino acid identity (AAI) values were calculated using the scripts ani.rb (with the options –win, 1,000; –step, 200; –len, 700; –id, 70) and aai.rb (with the options –len-fraction, 0.8; –id, 20), respectively, from the enveomics package v1.4.4 (16).

**TABLE 1:** Statistics summary of the coassembled and reassembled USCα genomes^*a*^

CheckM output	Beijerinckiaceae bin from coassembly	USCα AHI genome from reassembly
Marker lineage	o__Rhizobiales (UID3654)	o__Rhizobiales (UID3654)
No. of genomes	92	92
No. of markers	481	481
No. of marker sets	319	319
0 copies (missing)	**36**	**32**
1 copy	**444**	**449**
2 copies	**1**	**0**
3 copies	0	0
4 copies	0	0
≥5 copies	0	0
Completeness (%)	**90.56**	**91.64**
Contamination (%)	**0.31**	**0.00**
Strain heterogeneity (%)	0.00	0.00
No. of unique markers (of 43)	42	42
No. of multicopy markers	0	0
Insertion branch UID	UID3666	UID3666
Taxonomy (contained)	k__Bacteria;p__Proteobacteria;c__Alphaproteobacteria;o__Rhizobiales;f__Beijerinckiaceae	k__Bacteria;p__Proteobacteria;c__Alphaproteobacteria;o__Rhizobiales;f__Beijerinckiaceae
Taxonomy (sister)	Unresolved	Unresolved
GC content (%)	**59.1**	**59**
Genome size (Mbp)	**3.03**	**3.26**
Gene count	**3,388**	**3,928**
Coding density (fraction)	**0.82**	**0.81**
Translation table	11	11
No. of descendant genomes	3	3
Lineage		
GC content (%)		
Mean	60.6	60.6
SD	2.6	2.6
Genome size (Mbp)		
Mean	4.28	4.28
SD	0.13	0.13
Gene count		
Mean	3,861	3,861
SD	86	86

a Values that are different between the two draft genomes are marked in bold font.

**FIG 1:**
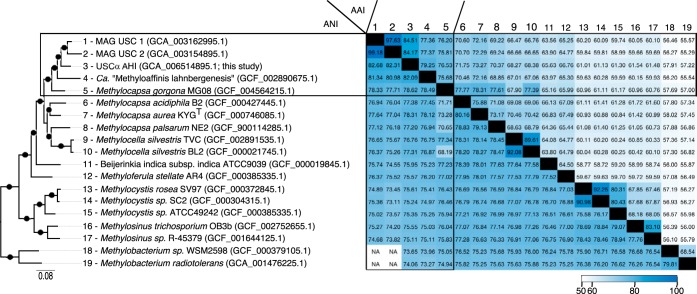
Genomic comparison between USCα AHI and genomes of methanotrophs within the *Beijerinckiaceae*. (Left) Phylogenomic tree constructed from 86 concatenated single-copy genes. The scale bar indicates the probability of substitution in amino acid residues. Filled circles indicate local support of 0.99 calculated using CAT approximation in FastTree v2.1.10 (included in Anvi’o v5.2). (Right) Matrix of pairwise ANI and AAI values ordered as indicated for the left panel. Black rectangles mark ANI and AAI values of USCα genomes. Color intensity indicates values between 55 and 100. NA, not available because fewer than 100 fragments (700 nt) shared an identity of >70%.

The USCα AHI genome belongs within the *Beijerinckiaceae* (Fig. 1) and possesses a 416-nt-long 16S rRNA gene that is 98.1 to 98.6% similar to published USCα 16S rRNA genes (10, 17). Its *pmoA* and *pmoB* genes match 99.7 to 100% with DNA and RNA sequences previously reported from AHI that were phylogenetically determined as the high-affinity form for CH_4_ oxidation (1). USCα AHI is able to assimilate C from CH_4_ and from CO_2_ via the serine cycle, the reductive glycine pathway, and the Calvin-Benson-Bassham cycle. USCα AHI can utilize various carbon sources via the pentose phosphate and Entner-Doudoroff pathways, including acetate in its tricarboxylic acid (TCA) cycle, although the acetate transporter gene (*actP*) is absent. The [NiFe] group 1h hydrogenase for H_2_ metabolism is also present.

### Data availability.

The draft genome sequence of USCα AHI has been deposited at NCBI GenBank under the accession number VDMG00000000 (BioSample number SAMN11877018 and BioProject number PRJNA545288). The version described in this paper is VDMG01000000. The raw reads of 21 metagenomes have been deposited at the NCBI Sequence Read Archive under the accession number SRP047512 (4).
